# Inhibition of Proliferation and Induction of Apoptosis by Gamma‐ or Delta‐Tocotrienols in Human Colorectal Carcinoma Cells

**DOI:** 10.1155/bmri/4421336

**Published:** 2025-09-30

**Authors:** Ali Qusay Khalid, Saatheeyavaane Bhuvanendran, Kasthuri Bai Magalingam, Premdass Ramdas, Ammu Kutty Radhakrishnan

**Affiliations:** ^1^ Food as Medicine Research Strength, Jeffrey Cheah School of Medicine and Health Sciences, Monash University Malaysia, Bandar Sunway, Malaysia, monash.edu.my

**Keywords:** apoptosis, cell cycle arrest, cell proliferation, colorectal cancer, flow cytometry, gene expression, tocotrienols

## Abstract

Colorectal cancer (CRC) remains a significant global health concern, necessitating the exploration of novel therapeutic approaches. This study investigated the anticancer effects of *γ*‐tocotrienol (*γ*T3) and *δ*‐tocotrienol (*δ*T3) on the human CRC cell lines HCC2998, HCT116, SW48 and Caco2. The cytotoxic effects were evaluated via cell viability assays, gene expression analysis and flow cytometry–based apoptosis detection. The results demonstrated that both *γ*T3 and *δ*T3 exhibited potent antiproliferative activities across all cell lines, with *δ*T3 generally showing lower IC_50_ values. Gene expression analysis revealed cell line–specific responses, with HCT116 cells demonstrating significant upregulation of apoptosis‐related genes, particularly in response to *γ*T3 treatment. Flow cytometry confirmed the apoptosis‐inducing capabilities of both compounds, with effects intensifying from 24–48 h of treatment. The response of HCT116 cells was the most pronounced, especially in response to *δ*T3 treatment. Both *γ*T3 and *δ*T3, after 48 h of treatment, induced significant G_1_ phase cell cycle arrest in HCC2998 cells, with *δ*T3 exhibiting a more pronounced suppression of S phase progression. These findings contribute to the growing evidence supporting the potential of T3 as a therapeutic agent for CRC, highlighting its ability to inhibit proliferation and induce apoptosis in multiple CRC cell lines. Further research is warranted to elucidate the precise mechanisms of action and evaluate the in vivo efficacy of these compounds.

## 1. Introduction

Colorectal cancer (CRC) is a major global health burden, ranking among the most commonly diagnosed malignancies and leading causes of cancer‐related deaths worldwide [1]. Although treatment modalities such as surgery, chemotherapy and radiotherapy have improved survival rates, the therapeutic outcomes are often compromised by drug resistance, recurrence and adverse side effects [2]. These limitations underscore the urgent need for novel, more effective and less toxic therapeutic strategies for CRC management.

In this context, natural compounds have gained increasing attention as promising agents for cancer prevention and treatment [3, 4]. Among them, tocotrienols (T3), a subgroup of vitamin E compounds found in palm oil, rice bran and annatto seeds, have shown considerable anticancer potential [5]. Particularly, the *γ*‐ and *δ*‐tocotrienol (*γ*T3 and *δ*T3) isomers have demonstrated superior anticancer activity compared to their tocopherol (TCP) counterparts in various preclinical cancer models, including colorectal carcinoma cell lines [6, 7]. These compounds exhibit pleiotropic anticancer effects, such as the inhibition of cell proliferation, induction of apoptosis and suppression of invasion and metastasis [8, 9].

The present study is aimed at investigating the antiproliferative and proapoptotic effects of *γ*T3 and *δ*T3 in human colorectal carcinoma cells. By evaluating their functional impact on cell viability and death pathways, this study provides insight into their therapeutic potential as targeted anticancer agents for CRC.

Mechanistically, *γ*T3 and *δ*T3 have been shown to modulate the expression of genes and proteins involved in key oncogenic pathways [7]. These include upregulation of proapoptotic mediators, downregulation of antiapoptotic factors and suppression of cell cycle progression through G_1_ arrest [10]. Additionally, T3 compounds downregulate inflammatory mediators such as Cyclooxygenase‐2 (COX‐2) and inhibit signalling cascades implicated in CRC progression, including the NF‐*κ*B, Akt/mTOR and Wnt/*β*‐catenin pathways [11–14]. This ability to concurrently target multiple cancer‐associated pathways highlights their therapeutic versatility.

Beyond their direct anticancer activity, *γ*T3 and *δ*T3 have also demonstrated chemosensitising properties, enhancing the efficacy of conventional chemotherapeutic agents like 5‐fluorouracil (5‐FU) and reducing their associated toxicity [15]. This dual functionality, as both standalone therapeutics and adjuvants, positions T3s as strong candidates in the development of combinatorial CRC treatment regimens.

Nevertheless, several challenges remain in translating T3 into clinical applications. These include limitations in bioavailability, the need to define optimal dosing and evaluation of long‐term safety and interactions with standard therapies [16]. While in vitro and in vivo studies consistently report selective cytotoxicity of T3 toward cancer cells with minimal effects on normal cells [17–19], comprehensive toxicity profiling and pharmacokinetic studies are still required.

Moreover, emerging data suggest that the anticancer efficacy of T3 can vary by cancer type, cell line and experimental conditions. In CRC, *γ*T3 and *δ*T3 consistently demonstrate the most potent anticancer activities among T3 isomers [20–23]. This variability further emphasises the need for targeted investigations into the specific molecular mechanisms underlying their actions in defined CRC models.

Taken together, this study contributes to the growing body of evidence supporting the anticancer role of T3 by specifically evaluating *γ*T3 and *δ*T3 in CRC cells. The findings provide mechanistic and functional insights into their ability to suppress tumour cell proliferation and trigger apoptosis. These results may inform the development of novel, multitargeted and less toxic therapeutic approaches for CRC.

## 2. Materials and Methods

### 2.1. Cell Culture Conditions

Four human CRC cell lines (HCC2998, HCT116, SW48 and Caco2) were obtained from the cell repository of International Medical University (IMU), Kuala Lumpur, Malaysia. Cells were maintained under standard culture conditions at 37°C in a humidified atmosphere with 5% CO_2_. HCC2998, HCT116 and SW48 cells were cultured in RPMI‐1640 medium, while Caco2 cells were maintained in DMEM, both supplemented with 10% fetal bovine serum (FBS) and 1% penicillin–streptomycin (Gibco BRL, Paisley, Scotland). Cells were detached using TrypLE Express and washed with phosphate‐buffered saline (PBS) prior to experimental procedures.

### 2.2. Determination of the Half‐Maximal Inhibitory Concentration (IC_50_)

For viability analysis, cells were seeded in 96‐well plates at a density of 5.0 × 10^3^ cells/well (HCC2998, HCT116, SW48) and 2.0 × 10^3^ cells/well (Caco2), followed by overnight incubation. The cells were treated with various concentrations (2–20 *μ*g/mL) of *γ*T3 or *δ*T3 (Davos Life Science Pvt. Ltd., Singapore; purity ≥ 97*%*). 5‐FU (Sigma–Aldrich Merck, United States) was used as a positive control. Treatments were applied for 24, 48 or 72 h, with untreated cells serving as negative controls.

Cell viability was assessed using the Cell Counting Kit‐8 (CCK‐8) assay (DOJINDO, Kumamoto, Japan), and absorbance was measured at 450 nm (reference 650 nm) using a Tecan microplate reader (Switzerland). The values were calculated using GraphPad Prism (v9.5.1) from normalised cell viability data. Additional reagents used included absolute ethanol (BDH, AnalaR), dimethyl sulfoxide (DMSO; Sigma–Aldrich) and UltraPure DNase/RNase‐free distilled water (QIAGEN, United States).

### 2.3. RNA Extraction and Gene Expression Analysis (Real‐Time Quantitative Polymerase Chain Reaction (RT‐qPCR))

Total RNA was extracted using the PureNA Fastspin Total RNA kit (Research Instruments Pte Ltd., Singapore), following the manufacturer’s protocol with slight modifications. Briefly, cells (2.0 × 10^6^) were lysed, and the lysate was processed through spin columns with multiple wash steps. RNA was eluted in 30–50 *μ*L of elution buffer and quantified spectrophotometrically.

Gene expression was assessed using the Luna Universal One‐Step RT‐qPCR Kit (New England Biolabs, United States), which integrates reverse transcription and quantitative PCR in a single‐tube format. The reactions were conducted with the following thermal cycling conditions: reverse transcription at 55°C for 10 min, initial denaturation at 95°C for 1 min, followed by 40–45 cycles of denaturation (95°C, 10 s) and extension (60°C, 30 s), with final melt curve analysis from 60 to 95°C.

Data were normalised to *GAPDH* as the internal control, and fold change in gene expression was calculated using the 2^−*ΔΔ*Ct^ method. A standard curve was generated using serial dilutions of human *GAPDH* cDNA. Genes selected for apoptosis pathway validation were chosen based on previous microarray findings (Table 1) [7].

**Table 1:** Common genes selected for RT‐qPCR validation studies.

**Primer**	**Forward sequence (5** ^′^ **–3** ^′^ **)**	**Reverse Sequence (5** ^′^ **–3** ^′^ **)**	**NM code**
*BIRC3*	GAT GTT TCA GAT CTA CCA GTG	GAA ATG TAC GAA CTG TAC CCT	NM_001165
*BIRC5*	CCA CCG CAT CTC TAC ATT CA	TAT GTT CCT CTA TGG GGT CG	NM_001012270
*CASP8*	GGA TGA TGA CAT GAA CCT GCT GG	TTG TTG ATT TGG GCA CAG ACT CTTT	NM_001080124
*CASP9*	CCG CTC GAG CTG CCT TAT CTT GCA CCC CA	AAG GAA AAA AGC GGC CGC GGG ACA CAA GTC ACT AGC CC	NM_001229
*PARP1*	ACT GCT AGC GGT AAT TGG GAG AGG TAG C	TGA GTC GAC TAG AGA AGG CAT CTG CAT TTT TAA TC	NM_001618
*GAPDH*	GTCTCCTCTGACTTCAACAGCG	ACCACCCTGTTGCTGTAGCCAA	NM_002046

### 2.4. Annexin V–FITC/PI Apoptosis Assay

Apoptosis induction was quantified using the Annexin V–FITC Apoptosis Detection Kit (BD Biosciences, United States) according to the manufacturer’s instructions. Cells (1 × 10^6^) were seeded in T‐75 flasks, incubated for 24 h and treated with *γ*T3 or *δ*T3 at their respective IC_50_ values for 24 or 48 h. Following treatment, cells were washed with cold staining buffer and resuspended in Annexin V binding buffer at 1 × 10^6^ cells/mL.

Each sample (100 *μ*L) was stained with 5 *μ*L Annexin V–FITC and 10 *μ*L propidium iodide (PI), incubated in the dark for 15 min at room temperature (25°C) and diluted with 400‐*μ*L binding buffer before flow cytometric analysis. FL1 and FL2/FL3 channels were used to detect Annexin V–FITC and PI fluorescence, respectively.

Early apoptotic cells were identified as Annexin V‐positive and PI‐negative, indicating externalisation of phosphatidylserine without loss of membrane integrity. Late apoptotic cells exhibited dual positivity for both Annexin V and PI, reflecting advanced stages of apoptosis accompanied by compromised membrane permeability. Necrotic cells were characterised by Annexin V‐negative and PI‐positive staining, consistent with the loss of membrane integrity without prior apoptotic signalling. In contrast, viable cells remained negative for both Annexin V and PI, indicating intact membranes and the absence of apoptotic features. This dual‐staining method allowed for discrimination between apoptotic and necrotic populations based on phosphatidylserine externalisation and membrane integrity.

### 2.5. Flow Cytometric Cell Cycle Arrest Analysis

HCC2998 CRC cells were seeded at a density of 1 × 10^6^ cells per T‐75 culture flask and incubated for 24 h under standard culture conditions (RPMI‐1640 supplemented with 10% FBS and 1% penicillin–streptomycin, at 37°C in a humidified 5% CO₂ atmosphere). Following attachment, cells were treated with complete media containing *γ*T3 or *δ*T3 at their respective IC_50_ concentrations. Untreated cells served as negative controls. After 48 h of exposure, cells were harvested for cell cycle distribution analysis.

Cell cycle profiling was performed using the BD Cycletest Plus DNA Reagent Kit (Becton, Dickinson and Company, United States), following the manufacturer’s protocol. A minimum of 5 × 10^5^ cells per condition was required for analysis. The procedure entailed the selective permeabilisation of cellular membranes using a non‐ionic detergent to release intact nuclei, followed by proteolytic digestion with trypsin to remove residual cytoplasmic and nuclear proteins. RNA was enzymatically degraded to prevent interference with DNA quantification, and nuclear chromatin was stabilised with spermine tetrahydrochloride.

Subsequently, isolated nuclei were stoichiometrically stained with PI, a DNA‐intercalating fluorochrome that binds quantitatively to double‐stranded DNA. Flow cytometric acquisition was carried out on a BD FACScan cytometer equipped with electronic doublet discrimination and an FL2 detector (585/42 bandpass filter), optimised for PI emission (peak detection range: 564–606 nm). A minimum of 10,000 events per sample was collected. DNA content histograms were analysed using FlowJo v10 and modelled with a Dean–Jett–Fox algorithm to quantify the percentage of cells in G_0_/G_1_, S and G_2_/M phases. The presence or absence of DNA aneuploidy (abnormal DNA stem lines) was assessed based on deviations in DNA index relative to the diploid control population.

## 3. Results

### 3.1. Cytotoxic Effects of *γ*T3 and *δ*T3 on Human CRC Cell Lines

In this study, the antiproliferative effects of *γ*T3 or *δ*T3 on HCC2998, HCT116, SW48 and Caco2 human CRC cells were evaluated in the presence of serum in the culture medium and in a dose‐ and time‐dependent manner. The graphical representations depicting the proliferation inhibition curves of the HCC2998, HCT116, SW48 and Caco2 cell lines are presented in Figure 1. These dose–response curves elucidate the cytostatic effects of the investigated compound on the aforementioned neoplastic cell populations. The IC_50_ values were obtained when HCC2998, HCT116, SW48 and Caco2 human CRC cells were cultured in the presence of *γ*T3 or *δ*T3 for 24, 48 or 72 h, the results of which are shown in Table 2. Concomitantly, the morphological alterations observed via high‐resolution microscopy are comprehensively documented in the Supporting Information, providing a visual corroboration of the cellular phenotypic changes induced by the experimental intervention.

Figure 1Quantitative assessment of the antiproliferative effects of *γ*‐tocotrienol (*γ*T3) or *δ*‐tocotrienol (*δ*T3) treatment on human colorectal adenocarcinoma cell lines at 24, 48, and 72 h posttreatment. (a) HCC2998. (b) HCT116. (c) SW48. (d) Caco2 cells. The graphical representations elucidate the differential inhibitory potency of *γ*T3 and *δ*T3 in comparison to the established chemotherapeutic agent 5‐fluorouracil (5‐FU), as measured by the half‐maximal inhibitory concentration (IC_50_) values.

**Figure:**
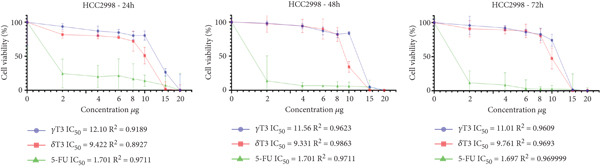
(a)

**Figure:**
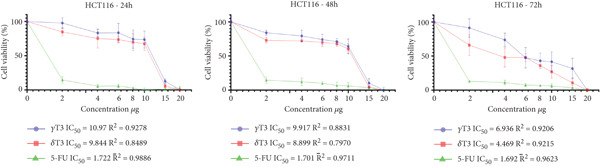
(b)

**Figure:**
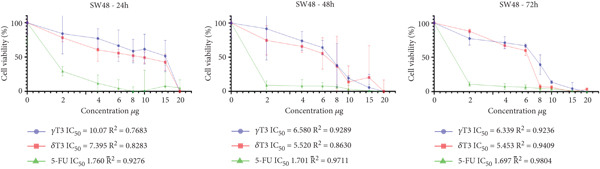
(c)

**Figure:**
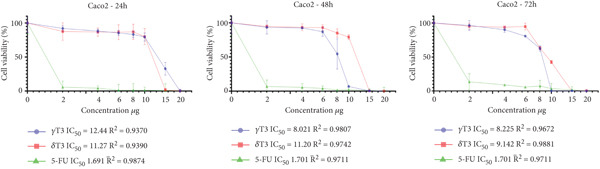
(d)

**Table 2:** The half‐maximal inhibitory concentration (IC_50_) values of *γ*‐tocotrienol (*γ*T3) and *δ*‐tocotrienol (*δ*T3) in human colorectal cancer (CRC) cells.

**Treatment**	**Cell line**	**IC_50_ (*μ*g/mL)**		
		**24** h	**48** h	**72** h
*γ*T3	HCC2998	12.10	11.56	11.01
	HCT116	10.97	9.91	6.93
	SW48	10.07	6.58	6.33
	Caco2	12.44	8.02	8.22

*δ*T3	HCC2998	9.42	9.33	9.76
	HCT116	9.84	8.89	4.46
	SW48	7.39	5.52	5.45
	Caco2	11.27	11.20	9.14

Abbreviations: *γ*T3: gamma‐tocotrienol; *δ*T3, delta‐tocotrienol; IC_50_, half‐maximal inhibitory concentration.

### 3.2. Gene Expression Analysis After *γ*T3 and *δ*T3 Treatment on Human CRC Cells

To elucidate the effects of *γ*T3 and *δ*T3 on the apoptotic pathway in CRC cells, a comprehensive analysis was conducted based on the apoptotic pathway identified through a previous scoping review [7]. RT‐qPCR was employed to examine the expression of selected genes involved in the apoptotic pathway (*BIRC3*, *BIRC5*, *CASP8*, *CASP9* and *PARP1*) in HCC2998, HCT116, SW48 and Caco2 cell lines. The validity of gene expression differences was determined by comparing the trends observed in RT‐qPCR with those identified in the scoping review. The analysis was represented in heatmaps, which revealed cell line–specific responses to *γ*T3 and *δ*T3 treatment (Figure 2).

Figure 2Quantitative analysis of apoptosis‐related gene expression in response to *γ*‐tocotrienol (*γ*T3) or *δ*‐tocotrienol (*δ*T3) treatment across multiple CRC cell lines. (a) HCC2998, (b) HCT116, (c) SW48, and (d) Caco2 cell lines were evaluated to elucidate the cell type–specific transcriptional responses to *γ*T3 and *δ*T3 treatment. Relative mRNA expression levels of *BIRC5*, *BIRC3*, *CASP9*, *CASP8*, and *PARP1* were quantified via reverse transcription quantitative polymerase chain reaction (RT‐qPCR) following 72 h of exposure to *γ*T3 or *δ*T3. Gene expression fold changes were calculated via the 2^–*ΔΔ*Ct^ method, with glyceraldehyde‐3‐phosphate dehydrogenase (*GAPDH*) serving as the endogenous reference gene for normalization. Differential gene expression is presented as the fold change relative to untreated controls, with values > 1.0 indicating upregulation and < 1.0 indicating downregulation.

**Figure:**
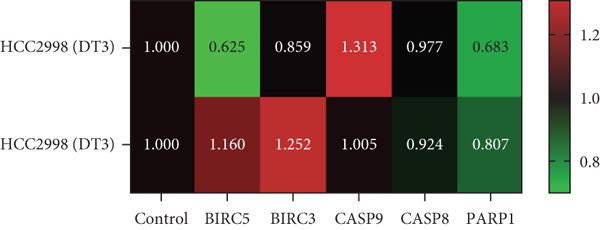
(a)

**Figure:**
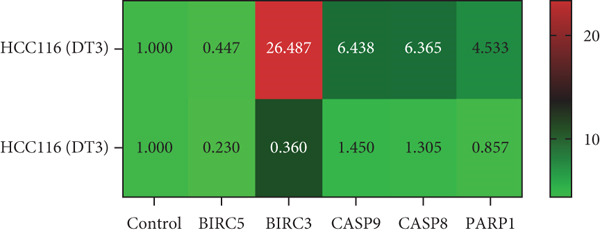
(b)

**Figure:**
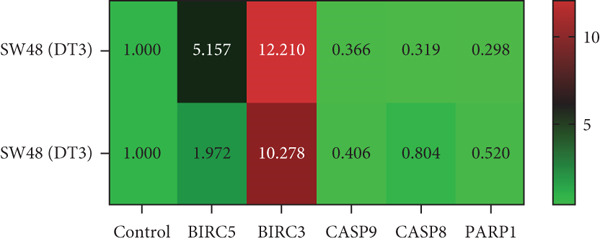
(c)

**Figure:**
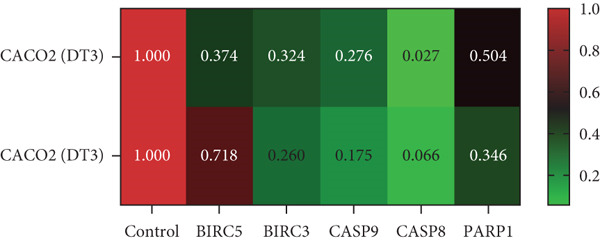
(d)

In HCC2998 and Caco2 cells, none of the genes examined presented more than a twofold change in expression. However, the following genes were significantly upregulated in HCT116 cells in response to *γ*T3 treatment: *BIRC3* (25 ± 4‐fold change), *CASP9* (5 ± 4‐fold change), *CASP8* (5 ± 3‐fold change) and *PARP1* (3 ± 5‐fold change). In contrast, *δ*T3 treatment of HCT116 cells resulted in the upregulation of only *BIRC3* (7 ± 3‐fold change). SW48 cells exhibited a distinct response pattern, with *γ*T3 upregulating *BIRC5* (4 ± 1‐fold change) and *BIRC3* (11 ± 2‐fold change), whereas *δ*T3 induced the upregulation of *BIRC3* (9 ± 2‐fold change) exclusively. Notably, these fold change values are relative to the control values and do not provide information on absolute gene expression levels.

### 3.3. The Apoptotic Effects of *γ*T3 and *δ*T3 on Human CRC Cells

Flow cytometry analysis of the apoptotic effects of *γ*T3 and *δ*T3 on human CRC cells revealed significant impacts across four cell lines: HCC2998, HCT116, SW48 and Caco2. Both *γ*T3 and *δ*T3 demonstrated potent apoptosis–inducing capabilities, with effects intensifying from 24–48 h of treatment (Figure 3). In the HCC2998 cell line, 90.28% of the control cells were healthy, which dramatically decreased to 41.68% and 37.25% after 48 h of *γ*T3 and *δ*T3 treatment, respectively. The HCT116 cell line exhibited the most pronounced response, particularly to *δ*T3. Starting from a control of 97.41% healthy cells, *γ*T3 reduced this percentage to 46.02% after 48 h, whereas *δ*T3 showed even greater efficacy, leaving only 39.52% healthy cells and inducing a remarkable 52.18% cell death. This represents the highest percentage of dead cells observed across all the cell lines and treatments. The SW48 cell line demonstrated moderate sensitivity to both treatments, with healthy cells decreasing from 92.38% in the control to 57.32% and 56.15% after 48 h of *γ*T3 and *δ*T3 treatment, respectively. Interestingly, *γ*T3 appeared slightly more effective at inducing necrotic cells in SW48 cells at 48 h (33.39%) than did *δ*T3 (29.67%). The Caco2 cell line showed a more balanced response between apoptosis and necrosis, with the percentage of healthy cells decreasing from 92.57% in the control to 58.88% and 53.57% after 48 h of *γ*T3 and *δ*T3 treatment, respectively. Notably, *δ*T3 was generally more effective than *γ*T3 in Caco2 cells, particularly in inducing early apoptosis at both time points. Across all the cell lines, the apoptotic effects of both *γ*T3 and *δ*T3 increased with treatment duration, which consistently resulted in lower percentages of healthy cells and higher percentages of apoptotic and dead cells at 48 h than at 24 h.

Figure 3Representative flow cytometry plots showing the apoptosis rates of (a) HCC2998, (b) HCT116, (c) SW48, and (d) Caco2 cells. The cells were subjected to *γ*‐tocotrienol (*γ*T3) or *δ*‐tocotrienol (*δ*T3) treatment for 24 or 48 h.

**Figure:**
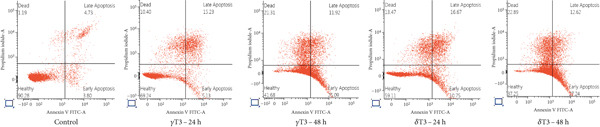
(a)

**Figure:**
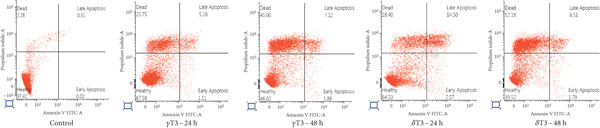
(b)

**Figure:**
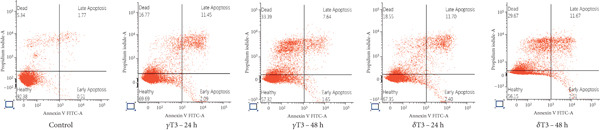
(c)

**Figure:**
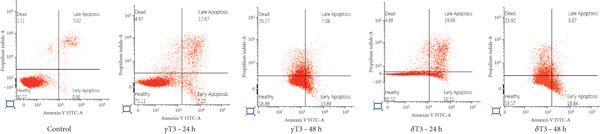
(d)

### 3.4. Cell Cycle Arrest Induced by *γ*T3 and *δ*T3 on Human HCC2998 Cell Line

The HCC2998 cell line was selected for this study due to its distinct genetic and phenotypic profile, representing a highly proliferative and metastatic model of CRC [24]. It is characterised by features that mimic late‐stage, aggressive CRC, including alterations in p53, *β*‐catenin and microsatellite stability (MSS) status [25, 26]. Moreover, it has been included in the NCI‐60 cancer cell line panel [27], which enhances its relevance and comparability in preclinical drug screening. Given its robust proliferative capacity, as reflected by a high baseline S phase fraction in DNA cell cycle analysis [28, 29], HCC2998 provides a sensitive platform to evaluate the cytostatic and proapoptotic effects of T3.

Flow cytometric analysis of DNA content following 48 h of treatment demonstrated that both *γ*T3 and *δ*T3 significantly altered the cell cycle dynamics of HCC2998 CRC cells. In untreated controls, the cell population exhibited a predominance in the S phase (51.2%), consistent with a high proliferative index. Treatment with *γ*T3 induced a redistribution characterised by an increase in G_1_ phase cells to 65.2% and a concomitant decrease in S phase to 32.1%, indicating a moderate induction of G_1_ phase cell cycle arrest. In contrast, *δ*T3 elicited a more profound arrest at the G_1_ checkpoint, as evidenced by a marked elevation in G_1_ phase to 76.6% and a substantial reduction in S phase to 20.4%. The G_2_/M phase distribution remained low in all conditions, suggesting that T3‐induced growth inhibition is primarily mediated through G_1_ phase arrest and suppression of DNA synthesis (Figure 4).

Figure 4Cell cycle analysis of HCC2998 cells treated with *γ*‐tocotrienol (*γ*T3) and *δ*‐tocotrienol (*δ*T3). Representative histograms of DNA content obtained by propidium iodide (PI) staining and flow cytometry illustrate the distribution of HCC2998 cells across cell cycle phases following 24‐h treatment. (a) Untreated control cells exhibit a high S phase population. (b) *γ*T3 treatment results in G_1_ phase enrichment and a reduction in S phase. (c) *δ*‐Tocotrienol (*δ*T3) induces a pronounced G_1_ phase arrest and a substantial decline in S phase. The histograms were modelled using a Dean–Jett–Fox algorithm, and percentages represent phase distributions derived from gated diploid populations.

**Figure:**
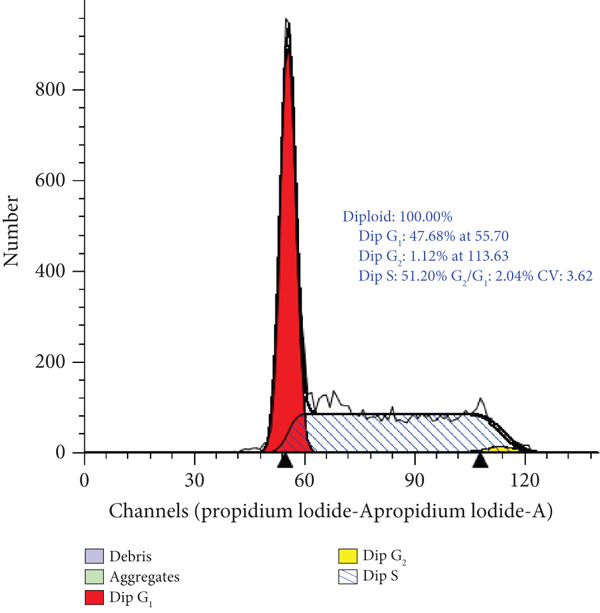
(a)

**Figure:**
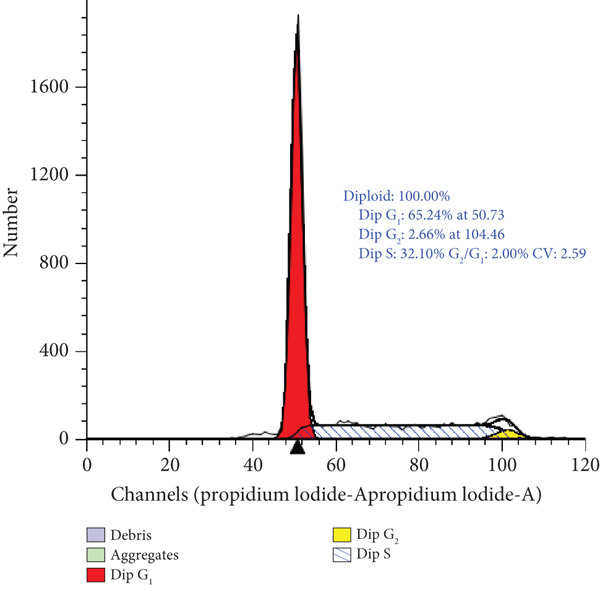
(b)

**Figure:**
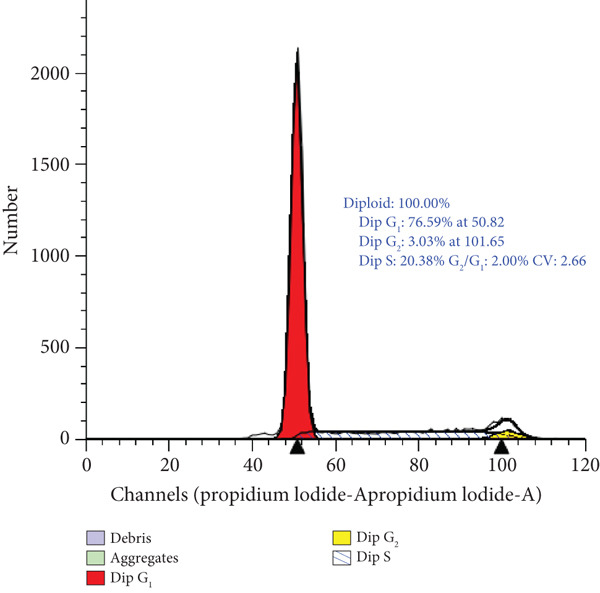
(c)

## 4. Discussion

Cancer is a heterogeneous disease with various molecular subtypes and genetic alterations [30, 31]. The selection of multiple cell lines ensures the representation of this diversity and increases the chances of capturing different responses to experimental treatments. The chosen cell lines, HCC2998, HCT116, SW48 and Caco2, exhibit distinct genetic alterations and metastatic potential, providing a comprehensive representation of CRC biology [32]. The isomers *γ*T3 and *δ*T3 have demonstrated significant antiproliferative properties across various cancer types, including CRC [10]. Their cancer cell–specific cytotoxicity, coupled with minimal impact on healthy cells, underscores their potential as therapeutic agents [33]. Temporal analysis of the intracellular *γ*T3 or *δ*T3 concentrations revealed distinct patterns across the cell lines. In HCC2998 cells, *γ*T3 levels modestly decreased over 72 h (12.10 *μ*g/mL at 24 h to 11.01 *μ*g/mL at 72 h), suggesting sustained intracellular presence. Conversely, the *δ*T3 concentrations fluctuated slightly (9.42 *μ*g/mL at 24 h, 9.33 *μ*g/mL at 48 h and 9.76 *μ*g/mL at 72 h), indicating potential cellular uptake and metabolic dynamics. The HCT116 cells demonstrated a more pronounced decrease in both *γ*T3 and *δ*T3 concentrations over time, with *γ*T3 levels decreasing from 10.97 *μ*g/mL at 24 h to 6.93 *μ*g/mL at 72 h. This trend suggests enhanced cellular uptake or metabolism in this cell line. Similarly, SW48 and Caco2 cells exhibited gradual reductions in the treatment concentration, albeit with varying kinetics [34].

The observed variations in IC_50_ values across different cell lines may be attributed to the heterogeneity of the CRC cells, potentially reflecting differences in genetic profiles, metabolic characteristics or expression of molecular targets relevant to T3 activity [35]. The consistently lower IC_50_ values for *δ*T3 than for *γ*T3 across all tested cell lines suggest that *δ*T3 may possess superior inhibitory potency against CRC cells. This observation aligns with previous studies indicating differential bioactivities among T3 isoforms and warrants further investigation into the structure–activity relationships and molecular mechanisms underlying these effects [36]. The contrasting concentration trends among the four cell lines highlight the importance of considering cell line–specific responses when evaluating the efficacy and mechanisms of action of anticancer agents.

Apoptosis is a tightly regulated cellular process orchestrated by several interrelated molecular pathways and regulatory proteins [37]. Two key protein families involved in the execution of apoptosis are the Bcl‐2 family and the caspases. The Bcl‐2 family of proteins, which are primarily localized to the outer mitochondrial membrane, consists of both proapoptotic (e.g., Bax and Bak) and antiapoptotic (e.g., Bcl‐2 and Bcl‐xL) members that collectively regulate mitochondrial outer membrane permeabilization (MOMP). Upon activation by apoptotic stimuli, proapoptotic proteins such as Bax facilitate the release of cytochrome *c* from the mitochondrial intermembrane space into the cytosol. Once in the cytoplasm, cytochrome *c* binds to Apaf‐1, forming the apoptosome complex, which subsequently recruits and activates initiator Caspase‐9, which is a critical step in triggering the caspase cascade that culminates in cell death [38]. Among the downstream targets of activated caspases is poly(ADP‐ribose) polymerase 1 (PARP1), a nuclear enzyme involved in DNA repair. During apoptosis, PARP1 is cleaved by effector Caspase‐3, thereby inhibiting DNA repair and conserving cellular energy to facilitate the progression of programmed cell death [37]. Previous studies have shown that *γ*T3 and *δ*T3 at low doses have antiproliferative and apoptotic effects on various types of cancer cells, including prostate, breast and stomach cancer cells (4–20 *μ*mol/L) [39–43].

The gene expression analysis conducted in this study offers further insight into the mechanisms by which *γ*T3 and *δ*T3 exert their anticancer effects. The observed upregulation of apoptosis‐related genes, especially in HCT116 cells, highlights the role of both isomers in modulating key molecular pathways involved in programmed cell death. Specifically, the significant upregulation of genes such as *BIRC3*, *CASP8*, *CASP9* and *PARP1* in response to *γ*T3 treatment points to the activation of intrinsic and extrinsic apoptotic pathways [44]. Caspases, particularly *CASP8* and *CASP9*, are known to be central players in the initiation and execution of apoptosis, with *CASP8* often being involved in the extrinsic pathway triggered by death receptors and *CASP9* functioning in the intrinsic mitochondrial pathway [45]. The upregulation of these genes suggests that *γ*T3 facilitates apoptosis through multiple mechanisms, enhancing its therapeutic potential by attacking cancer cells on multiple fronts.

Additionally, this study revealed that *δ*T3 induces the upregulation of *BIRC3* in HCT116 and SW48 cells, albeit to a lesser extent than *γ*T3 does. This gene is part of the inhibitor of apoptosis (IAP) family, which typically functions to block apoptotic processes [46]. The paradoxical upregulation of *BIRC3* may reflect a compensatory mechanism by which cancer cells resist the apoptosis‐inducing effects of *γ*T3 or *δ*T3 [47]. However, the overall proapoptotic environment created by the simultaneous activation of caspases and downregulation of antiapoptotic factors seems to override this resistance, leading to effective cell death [37].

The varying apoptotic responses among the cell lines suggest that the effectiveness of these treatments may depend on the specific characteristics of the cancer cells [48], with HCT116 cells appearing to be the most sensitive, particularly to *δ*T3. Both *γ*T3 and *δ*T3 activate the programmed cell death pathway by reducing the growth and inducing the apoptosis of human CRC cells. These results partly support previous observations regarding the inhibition of cell proliferation (Table 2). Interestingly, while both isomers demonstrated strong apoptotic effects, *δ*T3 consistently outperformed *γ*T3, particularly at the 48‐h mark. This differential potency aligns with previous research that highlighted the superior bioactivity of *δ*T3 compared with other isomers of T3 and TCP [5, 49, 50]. The time‐dependent increase in apoptosis from 24 to 48 h highlights the importance of sustained exposure for maximum therapeutic benefit, suggesting that longer treatment durations may be necessary to fully exploit their anticancer potential.

Importantly, although apoptosis (either early or late) was the dominant mode of cell death, a subset of cells, especially in SW48 and Caco2 lines, underwent necrosis, as indicated by Annexin V‐negative/PI‐positive staining. This necrotic population was more prominent at later time points, suggesting that prolonged exposure to T3 may trigger secondary necrosis or direct membrane disruption, potentially due to mitochondrial damage or overwhelming oxidative stress. From a therapeutic perspective, the occurrence of necrosis may have dual implications. While it contributes to tumour cell clearance, necrosis is often accompanied by the release of damage‐associated molecular patterns (DAMPs) that can provoke a local inflammatory response [51]. This warrants further investigation into the immunogenic consequences of T3‐induced necrosis in the tumour microenvironment, especially in immunocompetent models. Together, these molecular events confirm that *γ*T3 and *δ*T3 activate the intrinsic apoptotic pathway, especially regarding the observed increase in late apoptotic populations across the tested CRC cell lines.

The cell cycle arrest observed in T3‐treated HCC2998 cells underscores the cytostatic efficacy of *γ*T3 and *δ*T3, with the latter exhibiting a more robust inhibitory effect. *δ*T3‐mediated enrichment of cells in the G_1_ phase (76.6%) and depletion of the S phase population (20.4%) strongly indicate the disruption of G_1_‐to‐S phase transition, potentially through interference with cyclin D/E‐CDK4/6 regulatory axes or induction of CDK inhibitors such as p21^Cip1^ and p27^Kip1^. The comparatively modest G_1_ enrichment observed with *γ*T3 (65.2%) also supports its capacity to attenuate DNA replication and cell cycle progression, albeit to a lesser degree than *δ*T3. These findings align with previous reports highlighting T3‐induced modulation of cell cycle regulators and provide phenotypic confirmation of the antiproliferative effects observed in viability and apoptosis assays [10]. The lack of significant G_2_/M accumulation further suggests that the primary locus of action for both compounds is upstream of DNA synthesis, reinforcing their role as potent inhibitors of early cell cycle progression in CRC cells. The observed cell cycle redistribution and apoptosis induction following *γ*T3 and *δ*T3 treatment further reinforce the suitability of this cell line for mechanistic evaluations. Moreover, the differential potency observed between the two isoforms in G_1_ arrest emphasises the value of using a highly proliferative CRC model to reveal subtle phenotypic distinctions in treatment response.

Our findings are consistent with numerous studies in the field, which have shown that T3, particularly *γ*T3 and *δ*T3, induces apoptosis (either early or late) through multiple pathways, including the mitochondrial dysfunction pathway [52], extrinsic pathway [53], caspase activation [11] and PARP cleavage [54]. For example, Wada et al. reported that *δ*T3 has a direct antiproliferative effect on HT29 human colon carcinoma cells [55], whereas Abubakar et al. reported that *δ*T3 activated Caspase‐3, Caspase‐8 and Caspase‐9 and induced cell death in HT29 cells [56]. Similarly, Jang et al. reported that both *γ*T3 and *δ*T3 inhibited the proliferation of HCT116, HT29 and Caco2 CRC cells and induced apoptosis/autophagy [57]. The time‐dependent effects at both 24 and 48 h are particularly interesting and align with other studies, such as Shibata et al., who reported that *δ*T3 induced cell cycle arrest through CDK‐p21 modulation in DLD‐1 CRC cells [58].

These findings are consistent with the broader body of literature on T3, which has demonstrated its ability to induce apoptosis through a variety of mechanisms, including reactive oxygen species (ROS) generation and the inhibition of key survival pathways such as the NF‐*κ*B and PI3K/Akt pathways [59]. For example, previous studies have shown that *γ*T3 can downregulate the antiapoptotic protein Bcl–2 while upregulating proapoptotic proteins such as Bax, promoting cytochrome *c* release from mitochondria and activating caspases [60]. Additionally, T3 has been found to inhibit angiogenesis and metastasis by targeting pathways involved in cancer cell proliferation and migration, such as the Wnt/*β*‐catenin and mTOR pathways [61]. These multitargeting effects make T3 particularly attractive as anticancer agents, as it can disrupt several critical aspects of cancer cell survival and proliferation simultaneously.

The apoptotic effects likely involve the modulation of various molecular targets and signalling pathways, including cell cycle regulators, transcription factors, angiogenesis factors and metastasis‐related proteins [62]. Importantly, several studies have reported that T3 demonstrates superior selectivity for cancerous cells and is not toxic to noncancerous cells within the anticancer concentration range, which is a significant advantage for potential therapeutic applications. A study demonstrated that tocotrienol‐rich fraction (TRF) selectively inhibited proliferation and induced apoptosis in prostate cancer cell lines, evidenced by significant reductions in cell viability and colony formation, while exhibiting minimal cytotoxic effects on normal prostate epithelial cells, which only displayed modest viability decreases at elevated TRF concentrations [63]. In a clinical trial investigating the effects of *δ*T3 on pancreatic cancer, *δ*T3 selectively killed pancreatic tumour cells compared with normal cells at doses of 400, 600 and 800 mg/day [64]. These findings demonstrate the selective anticancer effect of T3 in a clinical setting while sparing normal, healthy cells.

While our study focused on *γ*T3 and *δ*T3 individually, Abubakar et al. reported synergistic antiproliferative effects when *γ*T3 or *δ*T3 was combined with Ficus species–derived alkaloids in HT29 cells, suggesting that T3 could enhance the efficacy of other anticancer agents [65]. The ability to induce apoptosis in multiple CRC cell lines, including those with different genetic profiles, indicates broad applicability. The differences in sensitivity between the various CRC cell lines to *γ*T3 and *δ*T3 are noteworthy and suggest that personalised approaches may be necessary when considering T3 for therapeutic use. In the present study, HCT116 cells demonstrated a pronounced susceptibility to both *γ*T3 and *δ*T3, particularly regarding the induction of apoptotic pathways, whereas SW48 and Caco2 cell lines exhibited comparatively attenuated responses. This variability could be attributed to differences in genetic mutations, metabolic activity or the expression of proteins involved in the uptake and metabolism of each compound. HCC2998 cells, which constitute a metastatic CRC model [24], also respond well to both *γ*T3 and *δ*T3, further indicating the potential of these compounds in treating advanced stages of the disease.

One of the significant contributions of this study is its demonstration of the dual role of T3 in both inhibiting proliferation and inducing apoptosis. These two processes are critical in the context of cancer treatment, as effective therapies must not only halt the growth of cancer cells but also actively promote their death. The ability of T3 to achieve both objectives highlights its potential as a standalone therapy or as an adjuvant to existing treatments. For example, previous research has shown that T3 can increase the efficacy of chemotherapeutic agents and dietary components by sensitising cancer cells to their effects, potentially allowing for lower doses of chemotherapy and reducing side effects [66].

Despite these promising findings, several challenges remain in translating the anticancer effects of T3 into clinical practice. One major obstacle is their bioavailability, as the absorption of T3 and TCP in the intestine varies from 20% to 80% of the total ingested amount, which is lower than that of other fat–soluble vitamins [36]. Various strategies are being explored to overcome these limitations, including the development of TRF formulations, nanoparticle delivery systems and combination therapies with bioavailability enhancers such as piperine [67, 68]. Additionally, more research is needed to determine the optimal dosing and treatment regimens for T3, as well as their long‐term safety and efficacy in humans. While in vitro studies provide valuable insights, in vivo studies and clinical trials are necessary to fully understand the therapeutic potential of T3 and to address issues such as dosage and interactions with other treatments.

## 5. Limitations and Future Directions

While this study provides substantial evidence for the antiproliferative and proapoptotic effects of *γ*T3 and *δ*T3 in CRC cells, it is not without limitations. First, although DNA content analysis via flow cytometry offered critical insight into cell cycle dynamics, other phenotypic endpoints were not evaluated in the current dataset. Additional assays are essential to delineate the full spectrum of T3‐mediated cytostatic and antimetastatic effects. Secondly, the current study focused primarily on cell‐based outcomes in a limited number of CRC lines, with HCC2998 serving as the primary validation model. While this line offers translational relevance, future work should involve broader panels to validate cell line–specific versus generalisable responses. Lastly, in vivo validations are warranted to confirm the therapeutic promise of *γ*T3 and *δ*T3 within the tumour microenvironment. Accordingly, future studies will incorporate phenotypic assays such as colony formation and 3D spheroid models to provide a more comprehensive view of T3‐induced functional impairments. These efforts will enhance translational relevance and contribute to a more holistic understanding of T3 efficacy in CRC therapy.

## 6. Conclusion

Collectively, our findings reinforce the growing body of evidence that *γ*T3 and *δ*T3 possess potent anticancer properties, primarily through the induction of apoptosis and modulation of cell cycle progression. The observed time‐dependent increase in apoptotic activity, coupled with selective cytotoxicity toward cancer cells, underscores their potential as targeted therapeutic agents for CRC. Notably, *δ*T3 exhibited superior efficacy in inducing late apoptosis and G_1_ phase arrest, highlighting its promise as a lead compound in future CRC treatment strategies. The emergence of necrotic subpopulations at later time points also raises important considerations regarding the balance between therapeutic efficacy and potential proinflammatory effects. As research advances, *γ*T3 and *δ*T3 may hold value not only as monotherapies but also in synergistic combinations with established chemotherapeutics or immunotherapies. Future investigations should aim to unravel the precise molecular mechanisms governing their dual cell death pathways, assess their immunomodulatory effects and validate these findings *in vivo* using CRC xenograft and orthotopic models to fully elucidate their therapeutic potential and translational relevance.

**Nomenclature**
5‐FU: 5‐fluorouracil
BIRC3: baculoviral IAP repeat containing 3
BIRC5: baculoviral IAP repeat containing 5
CASP8: Caspase 8
CASP9: Caspase 9
CCK‐8: Cell Counting Kit‐8
cDNA: complementary DNA
CRC: colorectal cancer
*C*t: threshold cycle
DAMPs: damage‐associated molecular patterns
DMEM: Dulbecco’s modified Eagle medium
DMSO: dimethyl sulfoxide
DNA: deoxyribonucleic acid
FITC: fluorescein isothiocyanate
*GAPDH*
: glyceraldehyde 3‐phosphate dehydrogenase
IAP: inhibitor of apoptosis
IC_50_
: half‐maximal inhibitory concentration
MOMP: mitochondrial outer membrane permeabilization
MSS: microsatellite stability
mTOR: mammalian target of rapamycin
NF‐*κ*B: nuclear factor kappa B
PARP1: poly(ADP‐ribose) polymerase 1
PBS: phosphate‐buffered saline
PCR: polymerase chain reaction
PI: propidium iodide
PI3K: phosphoinositide 3–kinase
RNA: ribonucleic acid
ROS: reactive oxygen species
RPMI: Roswell Park Memorial Institute
RT‐qPCR: reverse transcription quantitative polymerase chain reaction
T3: tocotrienols
TRF: tocotrienol‐rich fraction
TCP: tocopherols
*δ*T3: delta‐tocotrienol
*γ*T3: gamma‐tocotrienol

## Disclosure

This study was conducted as part of the doctoral dissertation of Ali Qusay Khalid, submitted in fulfillment of the requirements for the Doctor of Philosophy degree at the Jeffrey Cheah School of Medicine and Health Sciences, Monash University Malaysia.

## Conflicts of Interest

The authors declare no conflicts of interest.

## Funding

This study was supported by the Jeffrey Cheah School of Medicine and Health Sciences, Monash University Malaysia, 10.13039/501100021809, I–M010–STG–000195, and Design for Scientific Renaissance Sdn Bhd P–M010–CNI–000044.

## Supporting information


**Supporting Information** Additional supporting information can be found online in the Supporting Information section. The supporting figures provide microscopic examinations of human colorectal cancer cell lines (HCC2998, HCT116, SW48, and Caco2) treated with *γ*T3 or *δ*T3 at different concentrations for 24, 48, and 72 h. The images illustrate morphological changes compared to untreated controls, highlighting the dose‐ and time‐dependent effects of *γ*T3 and *δ*T3 at IC_50_ values. Figure S1: Microscopic examination of HCC2998 cells after (a) 24, (b) 48, and (c) 72 h. The first column shows the negative control, while the yellow circles indicate the IC_50_ values of treatments. The blue and orange rows represent *γ*T3 and *δ*T3 treatments, respectively, across different concentrations (2, 4, 6, 8, 10, 15, and 20 *μ*g/mL). The green row corresponds to the positive control (5‐FU). GT3: *γ*‐tocotrienol; DT3: *δ*‐tocotrienol; 5‐FU: fluorouracil. Figure S2: Microscopic examination of HCT116 cells after (a) 24, (b) 48, and (c) 72 h. The first column shows the negative control, while the yellow circles indicate the IC_50_ values of treatments. The blue and orange rows represent *γ*T3 and *δ*T3 treatments, respectively, across different concentrations (2, 4, 6, 8, 10, 15, and 20 *μ*g/mL). The green row corresponds to the positive control (5‐FU). GT3: *γ*‐tocotrienol; DT3: *δ*‐tocotrienol; 5‐FU: fluorouracil. Figure S3: Microscopic examination of SW48 cells after (a) 24, (b) 48, and (c) 72 h. The first column shows the negative control, while the yellow circles indicate the IC_50_ values of treatments. The blue and orange rows represent *γ*T3 and *δ*T3 treatments, respectively, across different concentrations (2, 4, 6, 8, 10, 15, and 20 *μ*g/mL). The green row corresponds to the positive control (5‐FU). GT3: *γ*‐tocotrienol; DT3: *δ*‐tocotrienol; 5‐FU: fluorouracil. Figure S4: Microscopic examination of Caco2 cells after (a) 24, (b) 48, and (c) 72 h. The first column shows the negative control, while the yellow circles indicate the IC_50_ values of treatments. The blue and orange rows represent *γ*T3 and *δ*T3 treatments, respectively, across different concentrations (2, 4, 6, 8, 10, 15, and 20 *μ*g/mL). The green row corresponds to the positive control (5‐FU). GT3: *γ*‐tocotrienol; DT3: *δ*‐tocotrienol; 5‐FU: fluorouracil.

## Data Availability

The data presented in this manuscript have been fully disclosed. However, the raw data are not publicly available due to laboratory confidentiality policies but can be provided by the corresponding author upon reasonable request, subject to institutional approval.
