# Supplementary material for: Inhibition of Proliferation and Induction of Apoptosis by Gamma‐ or Delta‐Tocotrienols in Human Colorectal Carcinoma Cells
Source: Biomed Res Int. 2025 Sep 30;2025:4421336. doi: 10.1155/bmri/4421336 (PMC12483303; doi:10.1155/bmri/4421336)
Supplement: Supplementary file 1 — Supporting Information Additional supporting information can be found online in the Supporting Information section. The supporting figures provide microscopic examinations of human colorectal cancer cell lines (HCC2998, HCT116, SW48, and Caco2) treated with γT3 or δT3 at different concentrations for 24, 48, and 72 h. The images illustrate morphological changes compared to untreated controls, highlighting the dose‐ and time‐dependent effects of γT3 and δT3 at IC50 values. Figure S1: Microscopic examination of HCC2998 cells after (a) 24, (b) 48, and (c) 72 h. The first column shows the negative control, while the yellow circles indicate the IC50 values of treatments. The blue and orange rows represent γT3 and δT3 treatments, respectively, across different concentrations (2, 4, 6, 8, 10, 15, and 20 μg/mL). The green row corresponds to the positive control (5‐FU). GT3: γ‐tocotrienol; DT3: δ‐tocotrienol; 5‐FU: fluorouracil. Figure S2: Microscopic examination of HCT116 cells after (a) 24, (b) 48, and (c) 72 h. The first column shows the negative control, while the yellow circles indicate the IC50 values of treatments. The blue and orange rows represent γT3 and δT3 treatments, respectively, across different concentrations (2, 4, 6, 8, 10, 15, and 20 μg/mL). The green row corresponds to the positive control (5‐FU). GT3: γ‐tocotrienol; DT3: δ‐tocotrienol; 5‐FU: fluorouracil. Figure S3: Microscopic examination of SW48 cells after (a) 24, (b) 48, and (c) 72 h. The first column shows the negative control, while the yellow circles indicate the IC50 values of treatments. The blue and orange rows represent γT3 and δT3 treatments, respectively, across different concentrations (2, 4, 6, 8, 10, 15, and 20 μg/mL). The green row corresponds to the positive control (5‐FU). GT3: γ‐tocotrienol; DT3: δ‐tocotrienol; 5‐FU: fluorouracil. Figure S4: Microscopic examination of Caco2 cells after (a) 24, (b) 48, and (c) 72 h. The first column shows the negative control, while the yellow circles i [file BMRI-2025-4421336-s001.pdf]

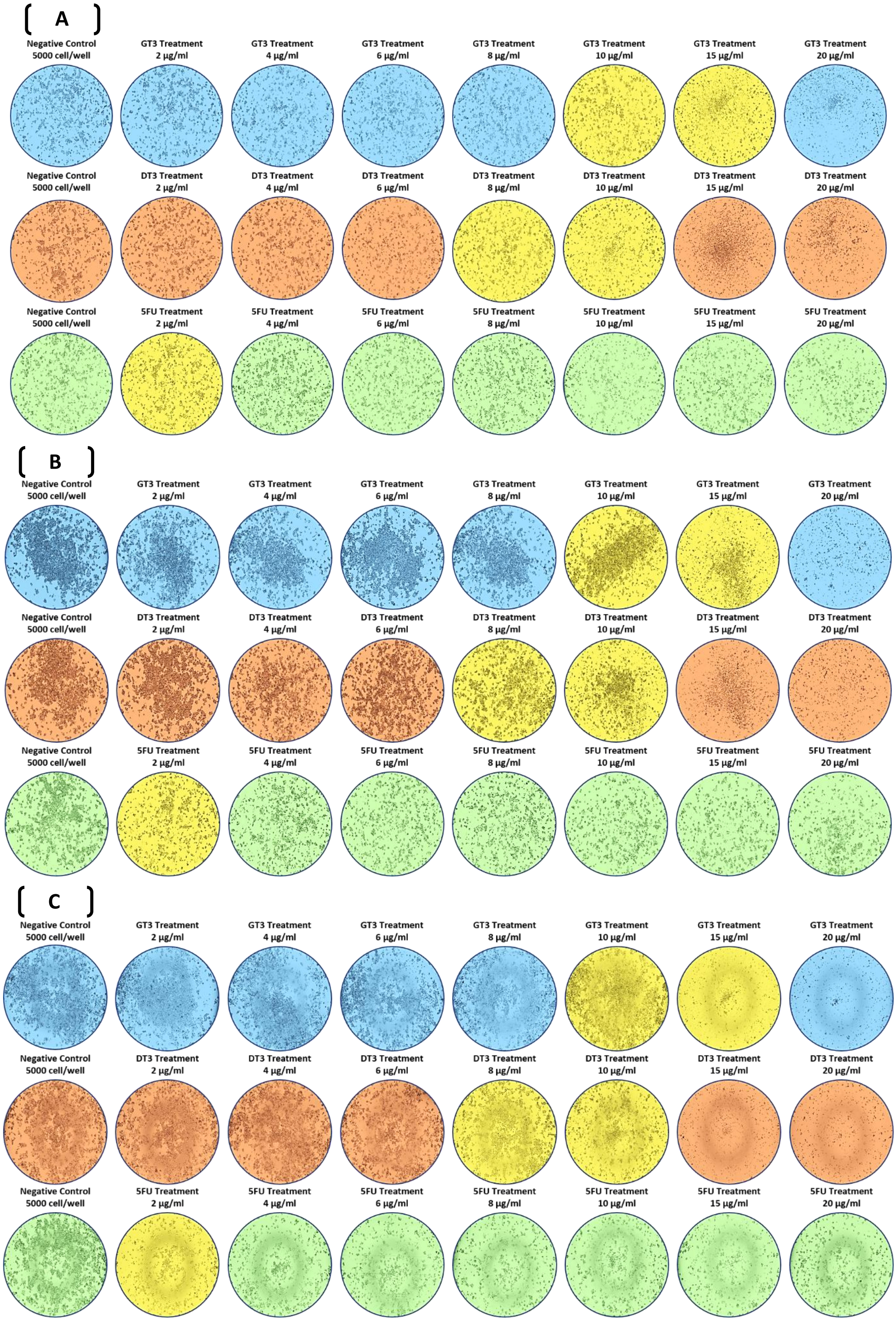

Supplementary Figure 1. Microscopic examination of HCC2998 cells after **(A)** 24 h, **(B)** 48 h, and **(C)** 72 h. The first column shows the negative control, while the yellow circles indicate the IC_{50} values of treatments. The blue and orange rows represent γT3 and δT3 treatments, respectively, across different concentrations (2, 4, 6, 8, 10, 15, and 20 µg/ml). The green row corresponds to the positive control (5-FU). GT3: γ-tocotrienol; DT3: δ-tocotrienol; 5FU: fluorouracil.

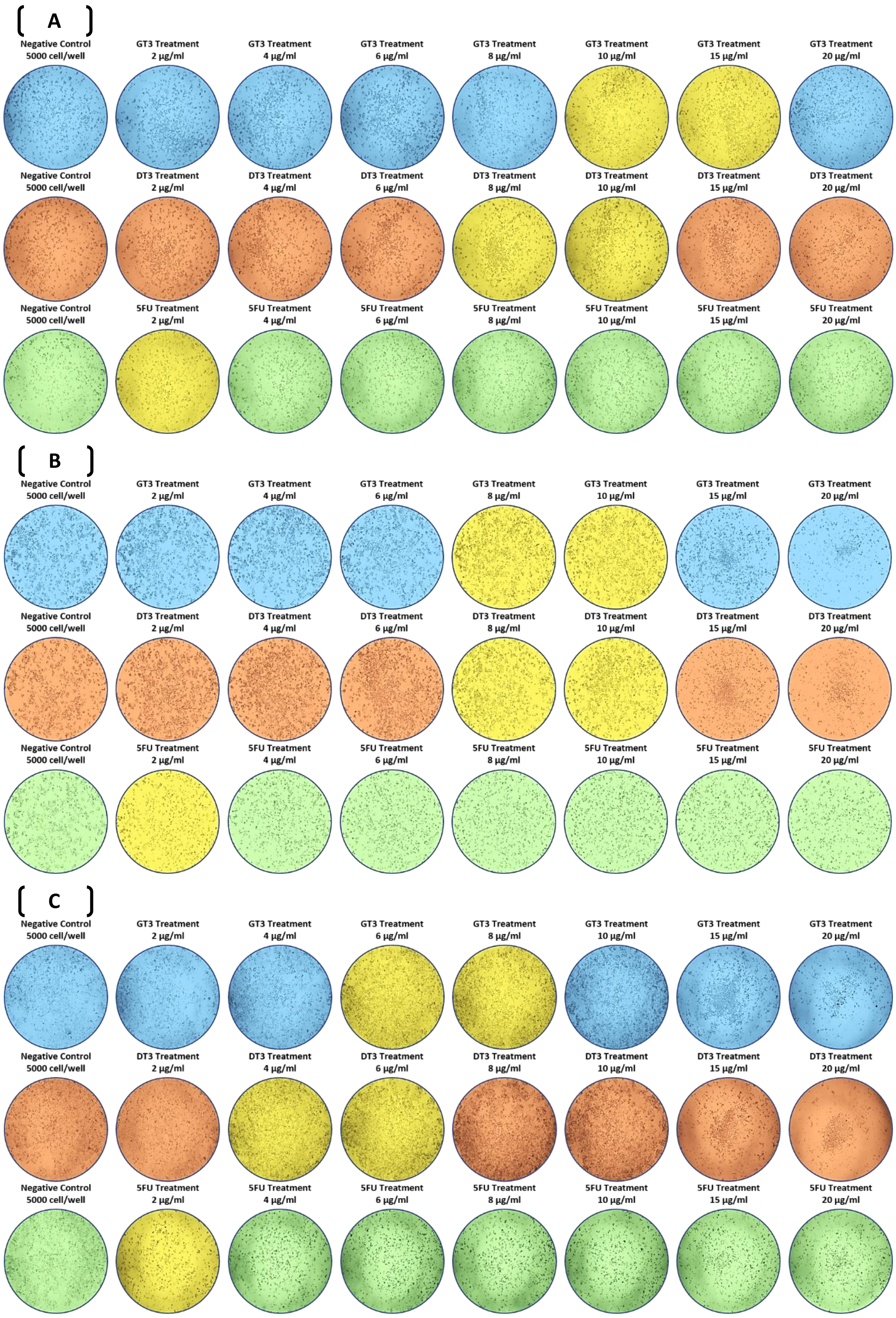

Supplementary Figure 2. Microscopic examination of HCT116 cells after **(A)** 24 h, **(B)** 48 h, and **(C)** 72 h. The first column shows the negative control, while the yellow circles indicate the IC_{50} values of treatments. The blue and orange rows represent γT3 and δT3 treatments, respectively, across different concentrations (2, 4, 6, 8, 10, 15, and 20 µg/ml). The green row corresponds to the positive control (5-FU). GT3: γ-tocotrienol; DT3: δ-tocotrienol; 5FU: fluorouracil.

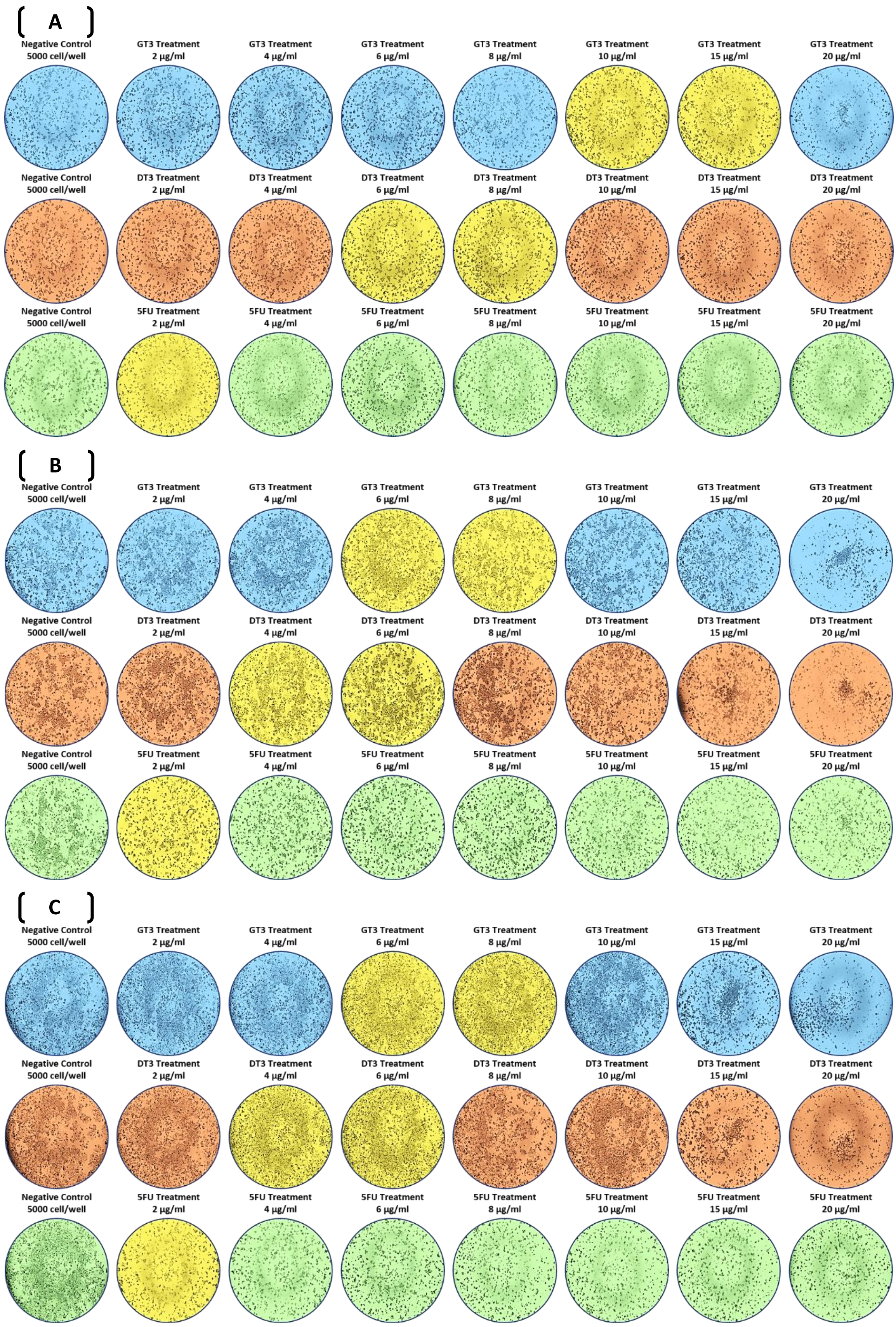

Supplementary Figure 3. Microscopic examination of SW48 cells after **(A)** 24 h, **(B)** 48 h, and **(C)** 72 h. The first column shows the negative control, while the yellow circles indicate the IC_{50} values of treatments. The blue and orange rows represent γT3 and δT3 treatments, respectively, across different concentrations (2, 4, 6, 8, 10, 15, and 20 µg/ml). The green row corresponds to the positive control (5-FU). GT3: γ-tocotrienol; DT3: δ-tocotrienol; 5FU: fluorouracil.

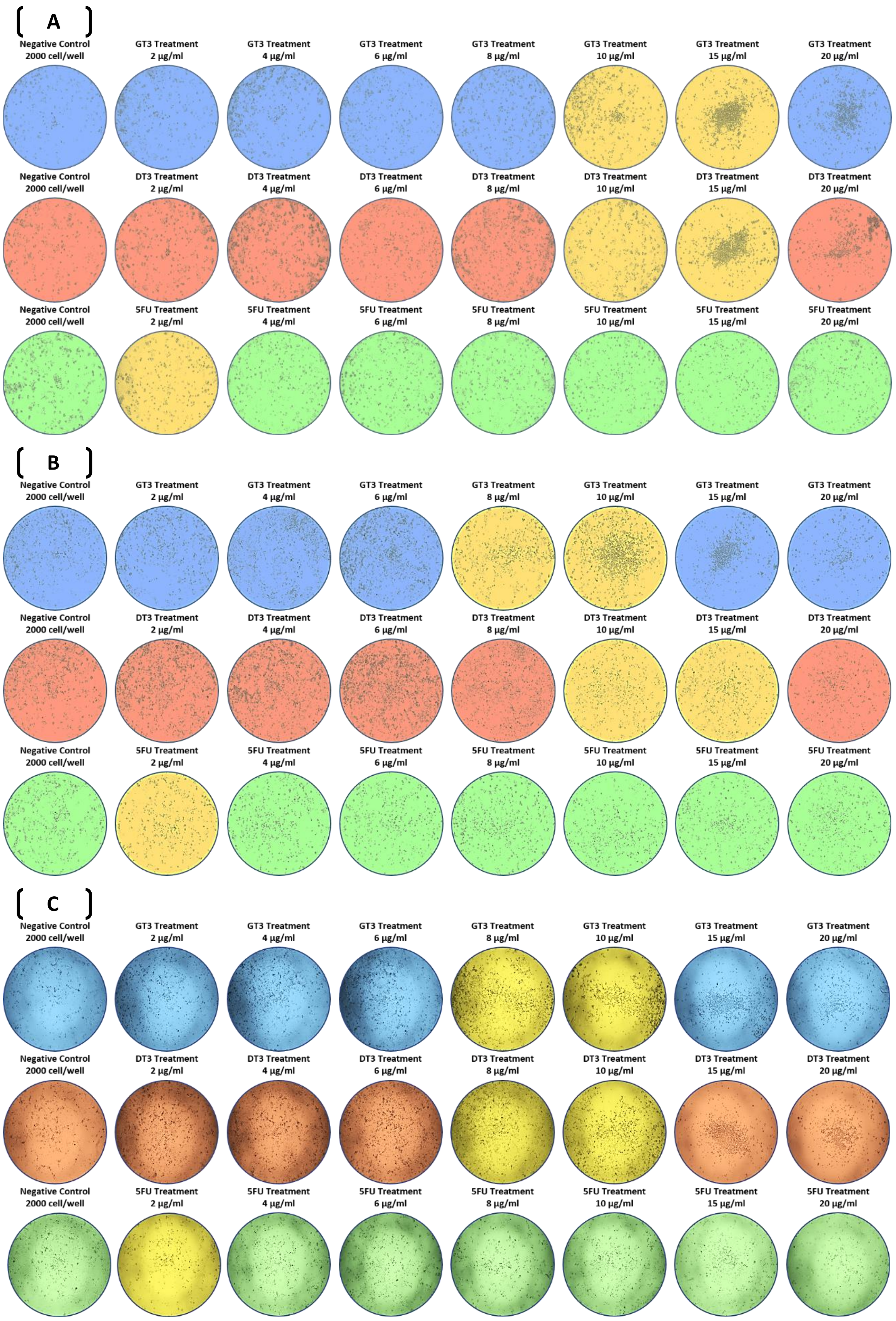

Supplementary Figure 4. Microscopic examination of Caco2 cells after **(A)** 24 h, **(B)** 48 h, and **(C)** 72 h. The first column shows the negative control, while the yellow circles indicate the IC_{50} values of treatments. The blue and orange rows represent γT3 and δT3 treatments, respectively, across different concentrations (2, 4, 6, 8, 10, 15, and 20 µg/ml). The green row corresponds to the positive control (5-FU). GT3: γ-tocotrienol; DT3: δ-tocotrienol; 5FU: fluorouracil.
